# ‘I Didn't Know, I Definitely Guessed.’ Exploring Pre‐Registration Podiatry Students' Approach to Identifying Dermatological Conditions in Different Skin Tones, a Mixed Methods Study

**DOI:** 10.1002/jfa2.70144

**Published:** 2026-04-02

**Authors:** Simon Otter, Faye Funnell, Alex Sykes, Kerry Ewins‐Strowger, Nadine Hemming, Deborah Whitham

**Affiliations:** ^1^ School of Health & Rehabilitation Science Health Sciences University Bournemouth UK; ^2^ Centre for Regenerative Medicine and Devices University of Brighton Brighton UK; ^3^ School of Education, Sport and Health Sciences University of Brighton Brighton UK

**Keywords:** decolonising education, health inequalities, mixed methods, podiatry, skin tone diversity

## Abstract

**Background:**

Research suggests that healthcare professionals find it more difficult to correctly diagnose dermatological conditions in the nonwhite patient demographic. People of colour experience higher rates of delayed and misdiagnosis, contributing to an increased mortality risk and increased health inequalities that remain widespread throughout the health care setting. This study aimed to investigate podiatry student's ability, confidence, approaches and perceptions in diagnosing dermatology pathologies in different skin tones.

**Methods:**

A mixed methods explanatory sequential design was undertaken with pre‐registration podiatry students from universities across South‐central England. Participants completed a validated pictorial multiple‐choice questionnaire comprising six images of either eczema or psoriasis in three different skin tone categories: light, medium or dark. Results were used to inform focus groups and a process of thematic analysis explored participants perceptions surrounding their diagnostic approaches and underpinning confidence.

**Results:**

The medium skin tone (Fitzpatrick groups III/IV) was associated with the most correct responses for psoriasis (69%) followed by light skin tone (Fitzpatrick groups I/II) with 48%. Psoriasis in darker skin tones (Fitzpatrick groups V/VI) received the least correct responses (3%). In eczema, results were more evenly spread with darker skin tones (Fitzpatrick groups V/VI), receiving a slightly higher percentage of correct diagnoses (39%). Qualitative analysis revealed two emergent themes: (i) reports on confidence and apprehension and (ii) limitations in education provision: each with a series of sub‐themes. Participants reported barriers to their diagnostic ability included an underrepresentation of dark skin tones in medical images and inadequate exposure to pathology on patients with dark skin tones.

**Conclusions:**

There was a notable lack of confidence in participants’ ability to correctly diagnose dermatological pathology, particularly in dark skin tones. This study addresses the research gap in podiatric health inequalities and pinpoints the associated educational shortcomings from the podiatry education perspective.

## 1 Introduction

Skin disorders represent a substantial portion of the global disease burden causing significant physical, psychosocial and financial impact [1, 2]. A recent European‐wide study highlighted a high prevalence of skin disorders, with 43% of participants reporting a dermatological complaint in the preceding 12 months [3]. Notably, skin complaints are reported to be the commonest reason people present to their general practitioner [4, 5]. In parallel, a high prevalence of foot disorders, including dermatological manifestations, was reported in a previous European‐wide study [6]. Presenting to a podiatrist enables a thorough examination of the lower limb and feet, highlighting the critical role of podiatrists in the timely assessment, identification, management and/or referral of dermatological manifestations [7]. However, a previous health needs assessment has suggested that greater training is needed in dermatology across primary care [8].

It has been widely reported that there are significant disparities in access to care, quality of care and diagnosis amongst patients from different racial, socio‐economic and ethnic backgrounds [9, 10]. Consequently, people with darker skin tones may receive a lower quality of care and are at a higher risk of morbidity and mortality [9, 11]. Reasons for this are complex, but include reduced access to care, limited training of professionals to confidently recognise skin lesions in people with darker skin tones and fewer visits to health professionals by this demographic [11, 12, 13, 14]. This is set against a background of a growing and increasingly diverse UK population, which brings with it a unique set of challenges [12].

Skin conditions can present differently, and pathological features can differ in people with darker skin tones [13, 14] For example, erythema seen in conditions such as eczema can present as red/purple with a burgundy undertone in people with darker skin tones [15]. Psoriatic lesions are typically harder to diagnose in people with darker skin tones appearing as dark brown, purple or greyish patches with silvery‐grey scales rather than the inflammatory patches seen on lighter skin [16, 17]. Psoriasis may also be both more severe with a greater reduction on quality of life in people with darker skin tones [16, 17]. Malignant pathologies in the skin, such as malignant melanoma, are less common in people with darker skin tones. However, the difficulties with early diagnosis means that people with darker skin tones may receive a confirmed diagnosis later compared to those with light skin tones leading to a higher rate of mortality [18, 19, 20].

Studies across professions demonstrate inconsistencies on how skin types are represented in education and training materials, often framed through a ‘white’ lens [21, 22, 23]. An audit of the A‐Z NHS website found just three images in people with darker skin tones compared to 61 images for light skin tones and only seven of the 75 pages described people with darker skin tones [24]. Considering online resources, 84% of physicians use ‘Google’ for professional purposes and images of people with darker skin tones were found to be underrepresented on this search engine [25]. This lack of exposure by medical professionals has led to skin tone bias [13, 26] and is thought to have contributed to a lack of confidence in diagnosis, misdiagnosis, delay in treatment or improper treatment across a range of dermatological complaints [11, 13, 27, 28, 29]. People with darker skin tones therefore face poorer prognoses and decreased survival rates compared to their white counterparts [30, 31]. These deeply embedded structural and systemic inequalities are evident throughout the different sectors of the healthcare setting [32]. This study seeks to explore the ability and confidence of final year podiatry students in diagnosing common skin conditions in people with darker skin tones.

## 2 Method

### 2.1 Study Design

A mixed methods explanatory sequential design was undertaken This design seeks to integrate quantitative and qualitative approaches to enhance understanding, uncover patterns and offer different perspectives of the issues highlighted in the study that neither approach could do alone [33, 34]. The two stages of the present study were as follows: Stage 1: A validated questionnaire provided quantitative data to inform. Stage 2: A qualitative component comprising a series of focus groups.

### 2.2 Ethics and Reflexivity

Ethical approval was granted by the University of Brighton, School of Sport and Health Science Research Ethics Committee (Ref 2022‐9784) and Health Sciences University Ethics Committee (Ref: HRS‐2024‐KshGz). Informed, written consent was gained prior to participation. The authors recognise that our position may have influenced our data analysis. All authors are podiatrists working in either education, private practise or public healthcare practise (NHS). To mitigate this potential bias, we received respondent validation to ensure that our interpretation of results was a true reflection of participants’ thoughts and perceptions. A pragmatic approach was used to focus on practical understandings of the real‐world concept under investigation alongside a critical research strategy. Together, these methodological choices have formed a strong connection to social justice inquiry in the literature [35].

### 2.3 Participants

A purposive nonprobability sample was taken from different groups of final year podiatry students from higher education institutions across south/central England. To mitigate potential cohort effects, data collection periods were undertaken in January–June 2022 and August‐December 2024. Participants chose to opt in via email from an internal university administrator to avoid potential bias/coercion from the research team. Participants included pre‐registration MSc and BSc final year students based on the inclusion/exclusion criteria (Table 1). To limit heterogeneity, participants had attended a similar number of taught sessions and completed a similar number of placement hours in their relevant institution.

**TABLE 1:** Inclusion/exclusion criteria.

Inclusion criteria Current podiatry student (final year) on either a pre‐registration or undergraduate podiatry course Aged 18 years or over Competency in English language to understand instructions and give full consent to participate Similar levels of clinical practise and academic exposure to peers in that cohort
Exclusion criteria Previous educational background/qualification in dermatology Past students who have already graduated/received an interim award from a podiatry course

### 2.4 Methodology

#### 2.4.1 Stage 1 Quantitative Data Collection

Following confirmation of written consent, a six‐item validated pictorial questionnaire [ 36—Supporting Information S1] was administered over a four‐week period. This contained six images of dermatological conditions (psoriasis and eczema) in three different skin tones based on the Fitzpatrick skin type scale [37]. For each question, there were six multiple choice answer options to choose from. For each image, participants were invited to choose and highlight the correct answer. To maintain consistency, each question had the same six answer options: cellulitis, psoriasis, erythema nodosum, scabies rash, eczema and none of these.

The pictorial questionnaire was emailed as a Word document to the course directors of each university. This was then individually forwarded to participants who completed the questionnaire in their own setting and returned to the research team. At the time of completion, participants were also invited to be part of a subsequent focus group.

#### 2.4.2 Stage 2—Focus Group Management

Following questionnaire completion, focus groups were held online via Microsoft (MS) Teams. Each focus group lasted approximately 60 min utilising a moderator and observer. To aid contextualisation and exploration of the questionnaire results, semi‐structured ‘jumping off’ questions were formulated, drawing key themes from the literature and questionnaire (Supporting Information S2). The correct answers to each question were revealed during the focus group, but without the revelation of the full study results, to encourage further discussion. With consent, the focus group was recorded using the MS Teams recording facility. This was then transcribed with the MS Teams live caption features and checked against the recording. The participants were offered the option of turning cameras off and notes were taken by the researcher of nonverbal communication such as nodding, facial expression and voice tone. Prior to commencing each focus group, further confirmation of consent was obtained.

## 3 Data Analysis

Categorical data from questionnaires indicated the total number of correct and incorrect answers for each skin condition across different skin tones. Data were tabulated and presented as a percentage for ease of comparison between groups. For qualitative data, transcripts were imported into a password‐protected university OneDrive for thematic analysis and analysed according to an interpretivist thematic paradigm [38] (Supporting Information S3). This paradigm assumes that social reality is shaped by human experience, is subjective and socially constructed according to our culture [39, 40]. To aid triangulation, all coding was completed independently by the authors. The research team then discussed and grouped the codes to construct themes and identified associated raw data excerpts. Disputes and disagreements regarding the construction of themes were resolved by further discussion. Verbatim quotes were used to highlight themes and participants were anonymised using culturally sensitive aliases assigned by the authors. To enhance the trustworthiness and credibility of the data, the authors sought respondent validation of the thematic findings and no recommendations for change were received.

### 3.1 Qualitative and Quantitative Component Integration

Integrating qualitative and quantitative data can strengthen the value of mixed methods research. In the present study, we sought to integrate qualitative and quantitative data throughout. Data integration took place at the design and methodological level together with analysis and reporting. Our design utilised an exploratory sequential design, whereby quantitative data sought to inform subsequent qualitative data generation. Importantly, the questionnaire used was previously designed using a methodologically integrated approach [36]. For ease of understanding, quantitative and qualitative findings are initially described separately in the results. In our study design the quantitative results informed the content and manner in which the qualitative component of the study was undertaken. Therefore, both elements were combined and embedded via the analysis considered in the discussion.

## 4 Results

### 4.1 Demographic Data

In total, 33 participants completed questionnaires, and 22 participants subsequently took part in one of four focus groups. We did not seek to detail individual demographics of those completing questionnaires to prevent inadvertent recognition of participants in relatively small student cohorts. Our sample group across focus groups consisted of 19 female and 3 males, with an age range between 20 and 52 years and several different ethnic groups represented.

### 4.2 Quantitative Data

The questionnaire sought to report two different inflammatory skin complaints (eczema and psoriasis) as depicted in different skin tones. For each condition, psoriasis was correctly identified more frequently (63% of participants) than eczema (48%). Results combining skin tone and dermatological condition are outlined in Table 2. The medium skin tone (Fitzpatrick groups III/IV) was associated with the most correct responses for psoriasis (69%) followed by light skin tone (Fitzpatrick groups I/II) with 48%. Psoriasis in darker skin tones (Fitzpatrick groups V/VI) received the least correct responses (3%). However, in eczema, results were more evenly spread with darker skin tones (Fitzpatrick groups V/VI) receiving a slightly higher percentage of correct diagnoses (39%).

**TABLE 2:** Number correct responses according to Fitzpatrick skin type and dermatological condition.

Skin type	I/II	I/II	III/IV	III/IV	V/VI	V/VI
Condition	Eczema	Psoriasis	Eczema	Psoriasis	Eczema	Psoriasis
Question number	1	6	4	3	5	2
% Correct responses	30%	48%	27%	69%	39%	3%

#### 4.2.1 Qualitative Data

Following data analysis, two overarching themes emerged that reflected participants experience and enable a deeper understanding of their experience. These themes were

- Diagnostic confidence and apprehension
- Limitations of education

Each theme was divided into a series of sub‐themes that detail varying aspects of participants experience (Table 3). Each theme and sub‐theme are presented and illustrated with verbatim quotes taken from participants transcripts. Culturally appropriate pseudonyms are used to represent participants.

**TABLE 3:** Overview of qualitative themes.

Over‐arching theme	Sub theme
Diagnostic confidence and apprehension	Hesitation and uncertainty
	Dermatological background knowledge
	Patient exposure/personal experience
Limitations in education provision	Limited representation different skin tones
	Perceived limitations in educators' knowledge
	Lack of drive to increase teaching diversity
	Issues of personal responsibility & role modelling

### 4.3 Overarching Theme: Diagnostic Confidence and Apprehension

#### 4.3.1 Hesitation and Uncertainly

This was a sensitive topic to discuss; participants were initially hesitant and lacked confidence in sharing their experiences in front of others and there were often periods of silence between conversations. This cautious behaviour was represented in body language and rapid eye contact between each participant to gauge who would speak first and break the silence. Many participants openly expressed a lack of confidence in diagnosing the dermatological conditions, especially those in the dark skin tone images. There was increased uncertainty in which diagnostic signs to look for in dark skin tones, leading to a sense of panic and anxiety among participants. In all groups, participants struggled to distinguish between what was the inflammatory skin condition, discolouration and skin tone. For example, Sami commented

I don't think I would really know what signs to look out for in darker skin tones if I'm completely honest

Most participants found the dark skin tone images more challenging, stating that these images did not have the obvious and clearly defined indicators that identified which dermatological condition was present in the image. Rosie reported

it [dermatological condition in image 2] wasn't immediately clear to me what it was so I think I probably guessed this one, unfortunately

Polly also commented in reference to image two of psoriasis on a dark skin tone

I feel like this one’s quite a tricky one in the sense that normally when you’re told about psoriasis, you’re looking for like the silvery plaques… here it doesn’t fit what we’re sort of typically told to look out for

Fatima agreed

Yeah, I put down the erythema nodosum one… and again this is always said that [in psoriasis] there’s always a silvery scaly thing which I didn’t think that one had.

Interestingly, this perceived deviation from the ‘typical’ clinical presentation of psoriasis led the participants to feel that their preconceived ideas around diagnostic certainty was under threat, Polly commented that they had been

lulled into a false sense of security

- Dermatological background knowledge

The apprehension and uncertainty outlined above indicated participants approached lesions in darker skin tones in a less constructive manner and were not always adequately considering the range of relevant differential diagnosis. Some participants used a process of elimination by trying to work out what they thought were typical features associated with the condition such as observed clinical features, location, familiar patterns, whether it was bilateral, skin vitality, redness, size and scaliness. Participants readily were able to describe core clinical signs, particularly in lighter skin tone, but often used simple terminology; for example, Paul noted

there’s a bit of redness, that is caused by scratching probably

Equally, references to lesion shape and location were used throughout, although sometimes in somewhat vague terms. Combining clinical signs and extent of lesions, participants were actively seeking to rule out common dermatological complaints. In relation to psoriasis images, Paul and Sarah reported

I think just scaly and a little bit of red, I think those are the two things

maybe not swollen the same way eczema would be

However, participants seemed to have limited understanding about why underlying ‘redness’ or ‘scaliness’ may be important in terms of overall diagnosis. In darker skin tones, participants found it more difficult to describe the clinical features present in the images, increasing their uncertainty and hesitation around the diagnosis of pathology, resulting in some simply guessing the diagnosis. Jan went on to note

I think it’s because it doesn’t look as bright [red]….because obviously it’s dark skin…it’s trickier

In dark skin images, participants often appeared confused, some unable to describe clinical signs accurately, which led them to then question their own confidence/ability to diagnose psoriasis in any skin tone, with Sarah admitting

It just made me think, I’m not really sure I can, even in white skin. I’m not sure I could reliably identify psoriasis

- Patient exposure/personal experience

For some participants, their own personal experience of a dermatological condition was perceived to increase diagnostic confidence interestingly throughout all the skin tones depicted. Halima explained in reference to image one of eczema on a light skin tone

I had one of my family member[s] having that so, um, I just put down as eczema because I have experienced it.

Similarly, Jan commented how lesion location could help with diagnosis in some complaints

I used to get eczema myself when I was younger, so locations are important

In reference to image five of eczema in a dark skin tone, Emily shared

I actually found this one fairly easy to diagnose, just ‘cause I’ve experienced eczema before,

Fatima agreed, highlighting

Someone said that their friend had [eczema], so that’s how they knew it was that

For others, the lack of similar external reference points reduced their diagnostic confidence.

Naomi, explaining one driving force behind her lack of confidence, in reference to image five of Eczema on a dark skin tone

[light skin] was my reference point because I’m white and I certainly struggled with having a reference point for the markers on the darker skin tone

Overall, participants considered lighted skin tones were often easier to diagnose, regardless of their own frames of reference, highlighting limitations around education and experience. Sarah summarised

I’ve never seen it [ referring to psoriasis in darker skin] but I’d imagine it’d be difficult

### 4.4 Overarching Theme: Limitations in Education Provision

This was a strong consistent theme around the lack of exposure to dermatology and minimal diverse exposure in their education, placement experiences and role modelling from educators.

- Limited representation of different skin tones

Underrepresentation of dark skin tone images at a classroom level was repeatedly observed. Tasha commented

if we’re in a lecture like I feel like we learn all with light skin tones and then [the educators] occasionally, I guess they’ll put a photo in of a dark skin tone.

Emily described this association of limited representation with the use of older educational resources

I think maybe a lot of the [lecture PowerPoint] slides as well were used from year to year. A lot of the slide shows that lecturers used is one slideshow that’s made, maybe four years ago, and that is using the same one. It’s not necessarily one that they’ve made now.

Participants also perceived printed literature had limited representation. Tara commented

*I don't think we really see darker skin tones in textbooks, at least not the ones I’ve seen*.

Underrepresentation of dark skin tone images was also observed by our participants on an international level, Naomi highlighting.

even on like skin cancer websites in America, here [UK], Australia, it was really, really, really, difficult [to find representation of dark skin tones] and even on public health websites… I wouldn’t feel that general healthcare professionals have the resources available to diagnose, umm, dermatological conditions in a variety of skin tones, let alone podiatrists!

Underrepresented lesions in skin of colour found in educational resources reiterated participants perception of the lack of importance and awareness of the gap in diverse representation.

- Perceived limitations in educators' knowledge

Our participants perceived their educators sometimes had an inadequate understanding of diagnosing dermatology conditions in different skin tones. Participants hypothesised that one factor behind this was associated with the educator's lack of exposure to a more diverse patient base. Lola commented

when I’ve treated people with darker skin tones, you also notice that like, the more our tutors don't always completely know cause its sometimes harder for them as well because of the demographic we work with.

Participants proposed that this limitation may arise from educators receiving a colonised curriculum themselves and therefore their teaching was a continuous cascade of this. They perceived that the academics themselves may have limited understanding and exposure to diagnosing dermatological conditions in dark skin tones. This meant that the students also often felt uncertainty in approaching educators around queries of diagnosis and skin of colour. Sarah reported

when I’ve treated darker skin tones…you also notice that like…the more like our tutors don’t always completely know

However, often participants also identified an apparent lack of confidence in educators diagnosing and managing skin presentations, even in relation to conditions being treated. Features of worsening health, for example, signs of infection were more at the forefront of educator's minds. Paul noted

I think cellulitis…they were quite interested in…then it’s let’s think about antibiotics

Some participants commented that the anatomical location of a dermatological lesion was also significant, with a perceived anatomical separation at the ankle. Toni reported

They’re [educators] not getting hands on… I’ve worked in community…. They don’t even touch the patient… don’t even ask them to roll their trousers up

Sarah recalled her placement experience

…when I was in particularly the MDT clinics, a lot of the focus was on the wound on the foot, and…if they had dressings on their legs, we’d still only undo the dressings up to that ankle. I’d feel like they would find something if they did

It is important to highlight there were areas of high‐quality education/role modelling that were recognised and praised by participants. Sarah recalled a positive experience of an educator's use of diagnostic language, differential diagnoses and explanation of referral pathways, albeit in relation to a ‘coincidental diagnosis’ of a potential melanoma:

There was one dermatology query and he [referring to the educator] showed on a chart…what it was and it looked like melanoma…and they did a lot…referring to dermatology…I feel like a lot of…good testing went on there…a lot of testing of the…size, measured in photos

- Personal responsibility and role modelling

Reflecting on self‐directed learning set by educators, participants perceived this often to be noninclusive of nonwhite skin tones. Although overlap is acknowledged, Tasha commented

we would go away and do self‐directed learning, but I think [the dermatology case‐studies given by educators] were actually all Caucasian… all those papers were based on Caucasian skin tones

Most participants believed it was the responsibility of the educators to teach them about diverse skin tones, rather than have personal responsibility in learning this themselves. It was reported that educators were obligated to teach them how to analyse, diagnose and treat dermatological conditions in different skin tones, as they would be the person/s of highest authority and experience. However, this was not their experience to date. This was a strong consistent sub‐theme that participants lacked exposure to dermatology coupled with minimal diversity exposure in their university education, placement experiences and role modelling from other podiatrists. Paul stated

I do feel like I haven’t had the best exposure to dermatology

Many talked about the lack of diversity and amount of dermatology pathology seen while on placement. Farah noted

we didn’t get much dermatology patients in our referral system

Others considered dermatological lesions might be a coincidental diagnosis with more focus on wounds and presence of infection being at the forefront of the clinician's minds. Some reported placement educators lacking confidence and not role modelling approaches to dermatology diagnosis, with Sarah highlighting

there was a lack of confidence in dermatology, I wasn’t encouraged by educators to palpate for temperature and feel for the quality

The discussion continued to consider educational responsibility and opportunities for improvement. The topic of placement opportunity received particular attention with suggestion that a dedicated dermatology placement, similar to some of their other dedicated placements, would prove helpful, with Paul suggesting

…like the vascular placement. Maybe if we had like a dermatology one…in the NHS or private

Participants also suggested and universally agreed (by nodding) that any placement in a more ethnically diverse area would be valuable. Proactive ideas to support and improve their ability and confidence around lesions in skin of colour included increased dermatology lectures in general, more diverse case studies, open book tests with medical history and using a ‘spotter science’ learning approach.

## 5 Discussion

This is the first study to explore insights of podiatry students in diagnosing skin lesions in different skin tones. In doing so, we have uncovered areas of practise/education to address that seek to enhance podiatrists' clinical ability and confidence.

The overall lowest rate of correct diagnoses was for psoriasis seen in the darkest skin type (Fitzpatrick IV/V). This may further reflect a general lack of familiarity with the symptoms of inflammatory skin conditions in darker skin, such as subtle grey/violaceous presentations of erythema, hyper or hypopigmentation and increased lichenification. The highest rate of overall correct diagnoses was demonstrated for psoriasis in lighter skin types, suggesting that clinical signs of psoriasis were viewed through a predominantly ‘light skin lens’ supporting the view that there was limited understanding of alternative presentations in much darker skin. In direct contrast, eczema had the highest rate of correct diagnoses in the darker skin tones (Fitzpatrick IV/V). Eczema often presents with papulation, lichenification and violaceous erythema favouring flexor surfaces in people with darker skin tones [41]. Reasons for these differences and the complexities therein are considered within the discussion.

Complexities around the definition of race and ethnicity exist and there are significant knowledge gaps in relation to skin conditions. Variability in visual presentation amongst diverse ethnic groups can be substantial and is often misinterpreted. Understanding of unique clinical phenotypes, intricate genetic and environmental factors and specific responses to therapy within a diverse population require more understanding [42]. The under‐reporting of race and ethnicity sometimes noted in published literature, together with an over‐representation of white skin in research and clinical trials, and differences in perceptions and understanding act further to compound the inequity [28]. Although limited in dermatological research, there are clear health disparities such as diagnostic delay, poor management and even poorer prognoses between Caucasian and non‐Caucasian individuals [43]. Our participants represented different ethnic groups, and while not fully representative of all podiatry students, there was a reported reliance on a limited set of symptoms, viewed primarily through a ‘light skin lens.’

Our findings are not unique to podiatry; a range of previous research highlights the paucity of skin of colour information and representation within teaching materials, medical textbooks and across learning interventions [21, 22, 23, 24, 25, 44, 45]. Indeed, as part of the development of the questionnaire used in the present study, the authors had to collapse Fitzpatrick skin type scales owing to a lack of validated images in different skin tones [36]. Most previous research has primarily focused on the confidence and ability of medical and pre‐medical curricular [46, 47, 48, 49]. In a recent study, Hutchinson et al. [14] reported trainee doctors received inadequate dermatology training, particularly in skin of colour. In total, 83% of participants felt uncomfortable managing dermatological conditions in these patient groups, with 100% misdiagnosing eczema and 80% psoriasis. A significant difference in the referral rate for white skin versus skin of colour (40% vs. 25%) was also reported. The podiatry curriculum has a broad spectrum of focus in dermatology, including inflammatory skin lesions [49], and regulated curriculum content includes conducting dermatological assessments, interpreting signs and symptoms of systemic dermatological disorders as they manifest in the lower leg [50]. Podiatrists, therefore, play an important role in diagnosis/managing skin complaints. Yet there remains little research about podiatry students' confidence and ability in diagnosing dermatological pathology in different skin tones.

Evidence suggests identifying and managing common skin complaints in skin of colour remains challenging for many health professionals. Biases in education and clinical training may lead to delays in diagnosis and treatment, resulting in greater morbidity and mortality [11, 23, 51]. For example, delayed diagnosis of wound infection or prolonged inflammation seen in inflammatory skin complaints such as psoriasis leads to anhidrosis, pruritus and hyperpigmentation [25, 28, 52, 53]. Equally, survival rates for individuals diagnosed with melanoma were significantly lower in those with skin of colour [54]. Reasons for this disparity remain complex, interrelated and include limited skin cancer education, a lower index of suspicion and increased socioeconomic barriers [43, 55]. Our findings would support the notion that the lack of diverse imagery depicting lesions in dark skin tones can result can lead to diagnostic error. The over‐representation of light and white skin tones means that the ability to recognise, diagnose and treat dermatological conditions in patients with skin of colour is impaired, effecting the quality of care and clinical practise. Our participants described that the most fundamental aspect of increasing their diagnostic confidence was greater exposure to pathology in patients with different skin tones. This aligns with the findings of Fourniquet et al. [56], where physicians and medical students' diagnostic confidence in brown and black skin was increased for eczema, psoriasis, melanoma and basal cell carcinoma following exposure to pathology on nonwhite skin tones.

How to improve education and training for podiatrists both pre‐ and post‐registration remains challenging. For example, commonly used internet sources are often limited in their representation of different skin tones [25]. As explored by our participants, their educators appeared limited by the inadequate access to diversity resources. As highlighted previously, educational resources and medical‐based research fail to adequately tackle the diversity concern [21, 23, 27]. Dermatology education within the podiatry curriculum and exposure to dermatology on placement needs attention, and opportunities in nonpodiatry settings should be explored to open new avenues of learning [57]. This supports the notion that greater exposure to diverse populations can improve confidence and ability [56]. Our findings also highlight the complexity surrounding diagnosis of skin complains in skin of colour, where limited experience in diagnosing conditions in different skin tones is reflected in teaching students to detect, diagnose and treat dermatological conditions often based on lighter skin tones [18, 44]. There has been an ongoing pedagogical imperative to ‘decolonize the curriculum’ which aims to balance the over‐representation of a Eurocentric/western epistemological lens towards a more diverse curriculum. This is part of a range of factors that impact curricula design, including the needs of diverse stakeholders, professional and regulatory requirements and financial constraints. Participants in the current study may be more aware of the educational imperative around curricula issues in a way that many qualified practitioners might not. The representations of diversity of skin tones in educational materials by incorporating inclusive resources such as Brown Skin Matters [58] and Mind the Gap [15] help accurately reflect our increasingly diverse society.

The strength of this study was the inclusion of different cohorts of students from several education institutions utilising a validated questionnaire as part of a mixed methods approach. Equally, we recognise that the participants we recruited were final year podiatry students and acknowledge our sampling may have influenced our interpretation of the data, impairing validity and variability, leading to increased bias [59, 60]. However, incorporating respondent validation into our research does provide an assurance of credibility and trustworthiness of data interpretation [61]. Including participants from more than one academic institution was intended to enhance generalisability. We sought to ensure diagnostic validity by taking the images of confirmed skin diagnoses (with permission) from The Primary Care Dermatology Society [62]. It could be argued that the images were not diverse enough. In terms of the questionnaire images, participants did not have access to the ‘real world’ cues available in a consultation such as medical history or indication of anatomical location for any of the images. Participants may have felt the need to tick each of the multiple‐choice answers at least once when assessing the images to ‘cover all bases’ and avoid misdiagnosis [63]. We note that although we have sought to include nonverbal responses to highlight agreement or disagreement within a group, this does not represent formalised data collection. This is an area in need of further consensus in the methodological literature [64].

In conclusion, our findings would suggest that there is a need to increase podiatry students' exposure to dermatology conditions and the different clinical presentations across the skin tones. This greater diversity would aid their diagnostic ability and confidence which in turn would negate potential consequences of misdiagnosis in an already disadvantaged population demographic.

## 6 Recommendations for Academic/Clinical Practise

1. Seek and encourage students to undertake additional external placement opportunities in more culturally and ethnically diverse settings.
2. Educators should work towards updating educational resources to incorporate more meaningful information, imagery and case studies on patients of colour.
3. Educational institutions continue to meaningfully commit to inclusion initiatives

## Author Contributions

**Simon Otter:** conceptualization (supporting), supervision (equal), writing – review and editing (equal), project administration (lead). **Faye Funnell:** methodology, investigation, data curation, formal analysis (equal), writing – original draft (equal). **Alex Sykes:** methodology, investigation, data curation, formal analysis (equal), writing – original draft (equal). **Kerry Ewins‐Strowger:** methodology, investigation, data curation, formal analysis (equal), writing – original draft (equal). **Nadine Hemming:** methodology, investigation, data curation, formal analysis (equal), writing – original draft (equal). **Deborah Whitham:** conceptualization (lead), writing – review and editing (equal), supervision (equal).

## Funding

The authors have nothing to report.

## Ethics Statement

Ethical approval was granted by the University of Brighton, School of Sport and Health Science Research Ethics Committee (Ref 2022‐9784) and AECC University Ethics Committee (Ref: HRS‐2024‐KshGz).

## Conflicts of Interest

The authors declare no conflicts of interest.

## Supporting information


Supporting Information S1

Supporting Information S2

Supporting Information S3

## Data Availability

The datasets generated and/or analysed during the current study are not publicly available due to participant confidentiality, at the behest of the ethics approval process.
