# Supplementary material for: ‘I Didn't Know, I Definitely Guessed.’ Exploring Pre‐Registration Podiatry Students' Approach to Identifying Dermatological Conditions in Different Skin Tones, a Mixed Methods Study
Source: J Foot Ankle Res. 2026 Apr 2;19(2):e70144. doi: 10.1002/jfa2.70144 (PMC13052160; doi:10.1002/jfa2.70144)
Supplement: Supplementary file 1 — Supporting Information S1 [file JFA2-19-e70144-s001.docx]

Supplementary file 1

PICTORIAL SURVEY

Instructions

The present questionnaire will show you six images of skin lesions. For each image, six diagnoses will be proposed (please, see the example below).

Skin lesion image

Option a

Option b

Option c

Option d

Option e

Option f

You are asked to tick and/or highlight the option that you think reflect the correct diagnosis. Only one answer is accepted (please, see the example below).

Skin lesion image

Option a

Option b

Option c


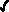


Option d

Option e

Option f

In case of multiple answers for a single image, the entire questionnaire will be excluded from the study.

THE PICTORIAL SURVEY WILL START ON THE NEXT PAGE

| Image 1: What is the most likely diagnosis for the skin lesion represented in this image? Please tick or highlight only one of the following options: |
| --- |
| 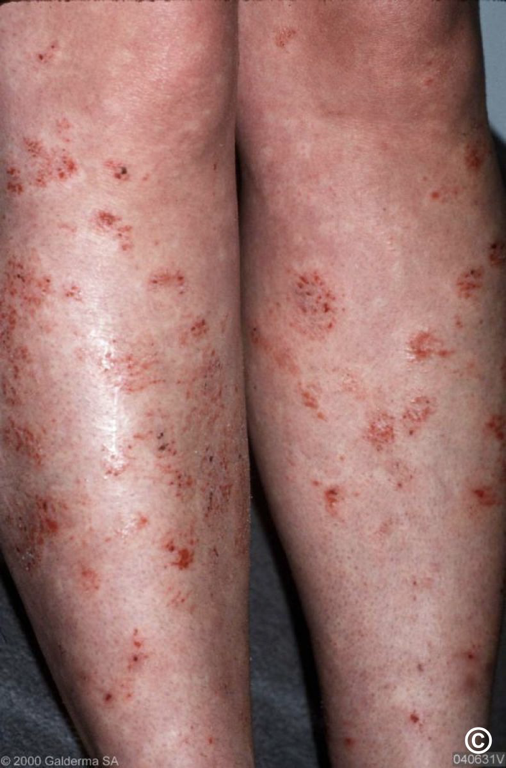 |
| Cellulitis  Psoriasis  Erythema nodosum  Scabies rash  Eczema  None of the above |

| Image 2: What is the most likely diagnosis for the skin lesion represented in this image? Please tick or highlight only one of the following options: |
| --- |
| 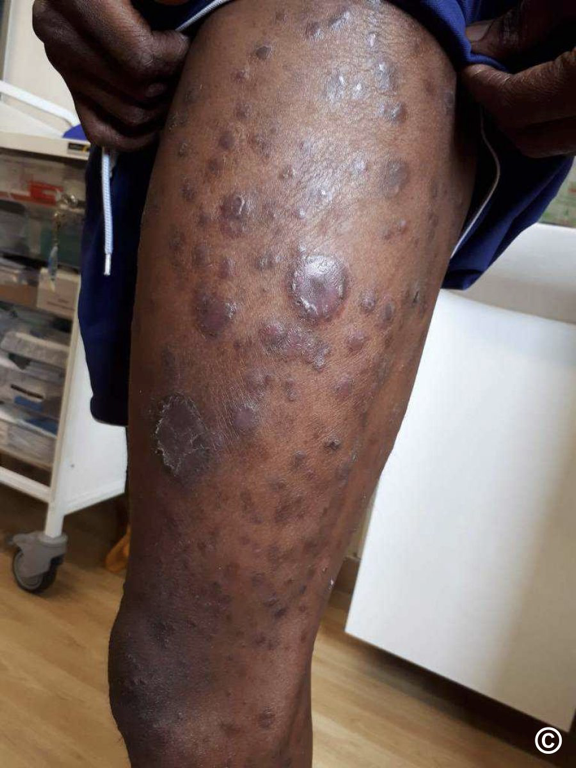 |
| Cellulitis  Psoriasis  Erythema nodosum  Scabies rash  Eczema  None of the above |

| Image 3: What is the most likely diagnosis for the skin lesion represented in this image? Please tick or highlight only one of the following options: |
| --- |
| 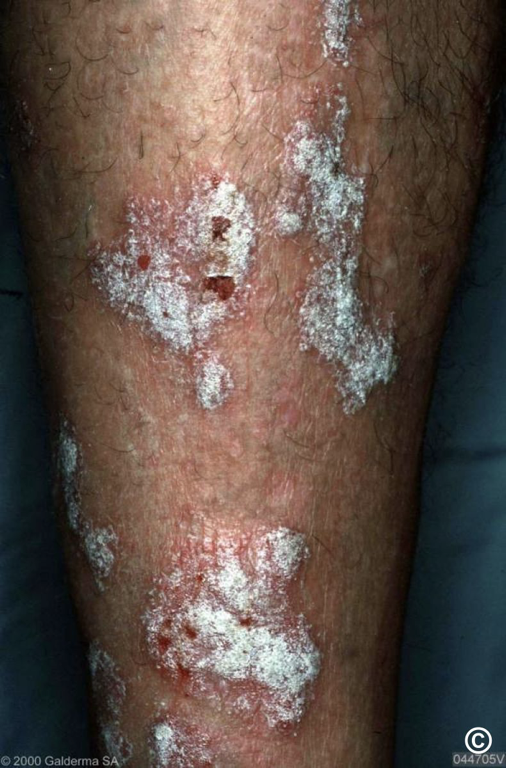 |
| Cellulitis  Psoriasis  Erythema nodosum  Scabies rash  Eczema  None of the above |

| Image 4: What is the most likely diagnosis for the skin lesion represented in this image? Please tick or highlight only one of the following options: |
| --- |
| 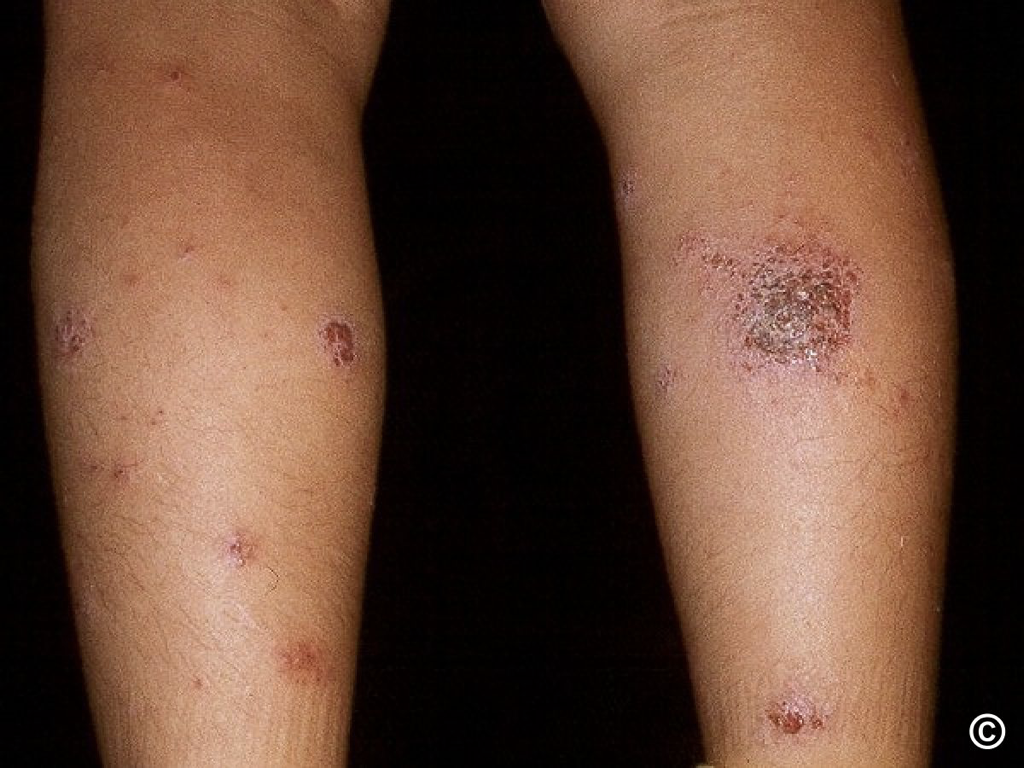 |
| Cellulitis  Psoriasis  Erythema nodosum  Scabies rash  Eczema  None of the above |

| Image 5: What is the most likely diagnosis for the skin lesion represented in this image? Please tick or highlight only one of the following options: |
| --- |
| 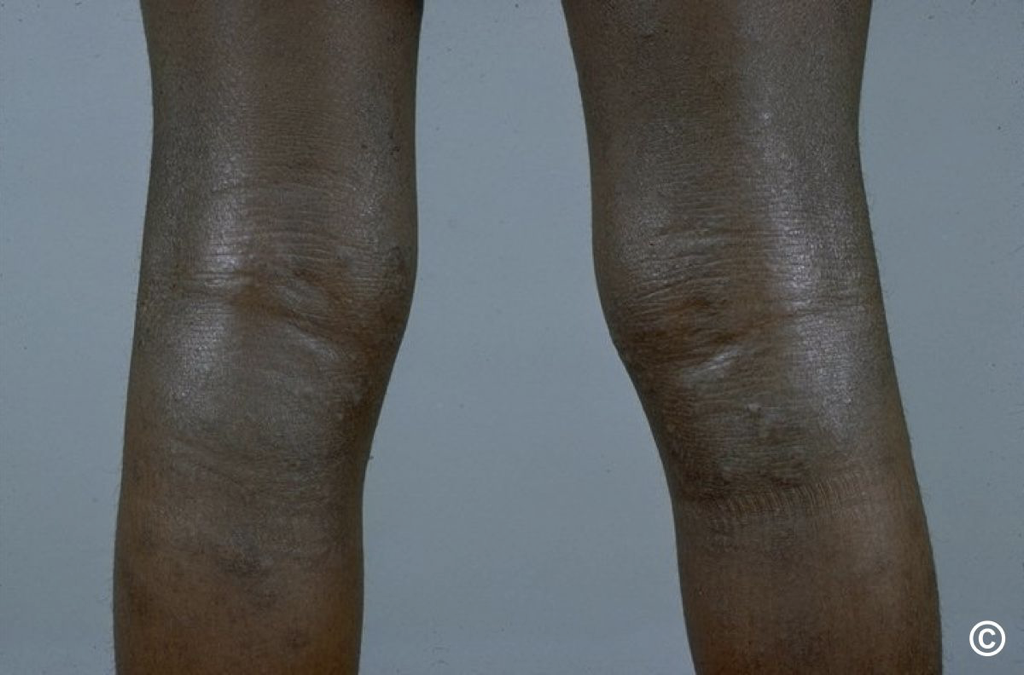 |
| Cellulitis  Psoriasis  Erythema nodosum  Scabies rash  Eczema  None of the above |

| Image 6: What is the most likely diagnosis for the skin lesion represented in this image? Please tick or highlight only one of the following options: |
| --- |
| 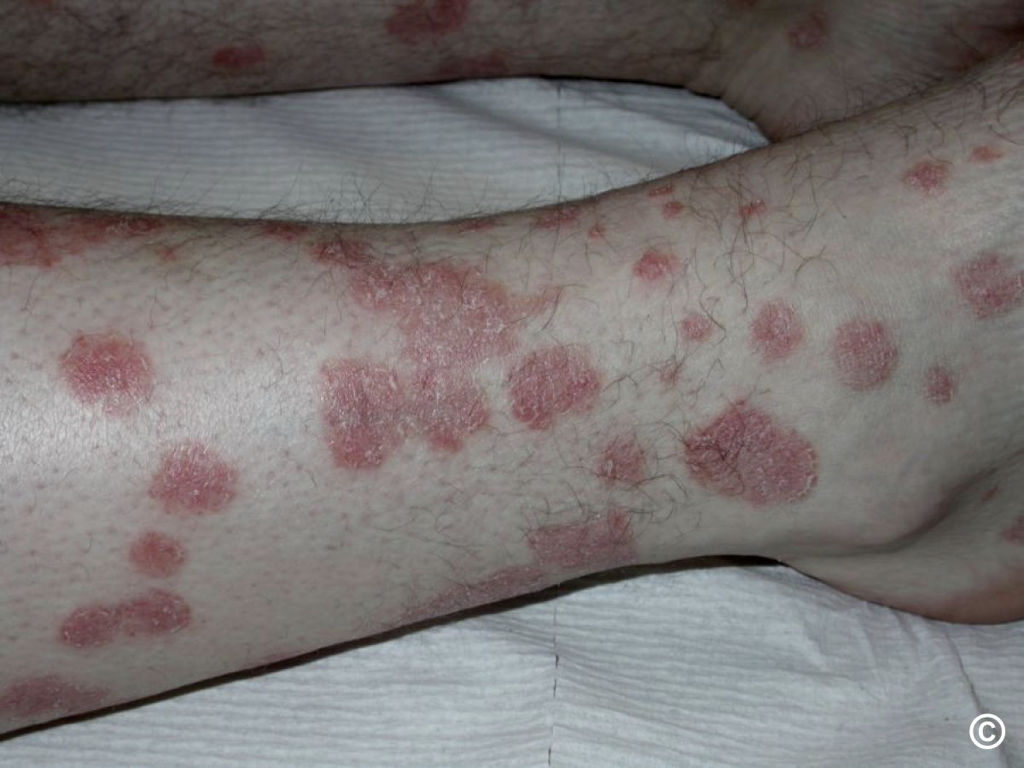 |
| Cellulitis  Psoriasis  Erythema nodosum  Scabies rash  Eczema  None of the above |

The pictorial survey is over.

Please save and rename this document as per your identification number and email it back to (Name and Email)

Should you wish to participate in the focus group to explore this topic further, please indicate your availability in the same email.

The focus groups will be conducted online via Microsoft Teams (more details will be provided via email).

We are grateful for your time and availability in the participation of our study.

Thank you
