# Supplementary material for: ‘I Didn't Know, I Definitely Guessed.’ Exploring Pre‐Registration Podiatry Students' Approach to Identifying Dermatological Conditions in Different Skin Tones, a Mixed Methods Study
Source: J Foot Ankle Res. 2026 Apr 2;19(2):e70144. doi: 10.1002/jfa2.70144 (PMC13052160; doi:10.1002/jfa2.70144)
Supplement: Supplementary file 2 — Supporting Information S2 [file JFA2-19-e70144-s003.docx]

Appendix 2- Semi-Structured Focus Group Questions

| Time expected (mins) | Description | Questions and prompts (if required) |
| --- | --- | --- |
| Welcome  0-5 | Welcoming participants and introducing research team  Reminding them we will be recording & prompting to turn on camera if they wish to do so, mention anonymity and confidentiality of the data  Ask participants to mute themselves when they are not speaking  Review the project aim | Terms for consistency  “As in the Participant information sheet your name will be unidentifiable to your input and the transcript will be anonymised and stored on the university OneDrive”  “Exploring Podiatry Pre-Reg Students Ability and Confidence to Diagnose Common Dermatological Conditions in Different Skin Tones” |
| START RECORDING | | |
| Diagnostic Approach  5-30 | Researcher will show images in the chat box in the order of the questionnaire  Reveal correct diagnoses | Jumping off question  For each image the question will be: “How did you approach diagnosis/ identifying clinical signs?”  Prompt: visual descriptors e.g. colour, size, shape, location, texture |
| Confidence in diagnosis and educational factors  30-55 | Firstly, the researchers will investigate the level of confidence in making diagnoses  Secondly the researchers will investigate the educational and/or social, clinical factors associated with the level of confidence in making diagnoses  How could we support podiatrists in their diagnosis of dermatological questions in non-white skin? | Jumping off Question  “Tell me about your confidence levels when diagnosing and detecting clinical signs across the different skin tones?”  Prompt: how do you know? Were you aware off differences in presenting features?  Jumping Off Question  Tell me about your own education and training- did you have access to non-white pictures?  Prompt: barriers to representation and health equalities, dermatology teaching improves confidence  Jumping off Question  Tell me about what helped your confidence and what you feel could improve your level of confidence in detecting clinical signs/ making diagnoses in different skin tones”  Prompt: curriculum, patient demographics and exposure, classroom resources, teaching skills/aids, what could the university do in preparation?  Jumping off Question  “What do you think would support you when making diagnosis in mixed skin tones”  Prompt: diagnostic language/ expectations used for dermatology e.g. red, erythema, rubor, pallor  CLOSING QUESTION: “Have we missed anything you would like to discuss/comment?” |
| STOP RECORDING | | |
| Conclusion and closing remarks  55-60 | Thank the participants  Any questions about the project |  |
