# Supplementary material for: ‘I Didn't Know, I Definitely Guessed.’ Exploring Pre‐Registration Podiatry Students' Approach to Identifying Dermatological Conditions in Different Skin Tones, a Mixed Methods Study
Source: J Foot Ankle Res. 2026 Apr 2;19(2):e70144. doi: 10.1002/jfa2.70144 (PMC13052160; doi:10.1002/jfa2.70144)
Supplement: Supplementary file 3 — Supporting Information S3 [file JFA2-19-e70144-s002.docx]

Appendix 2: Description of the processes undertaken by the authors for thematic analysis

| Stage of thematic analysis [38] | Steps taken by the authors |
| --- | --- |
| Familiarisation with the data | Authors individually corrected the automatic transcript of audio data by manual processes to ensure data was transcribed verbatim. The authors recorded their preliminary thoughts of the data during this process. Each author read the transcript several times. |
| Coding | Authors individually highlighted and noted specific and relevant features of the raw data to initiate the generation of codes. Authors also noted their own interpretation of the data where appropriate. Data was processed systematically and consistently. |
| Generating initial themes | Collectively the authors searched the codes for patterns and themes, this was done thoroughly, firstly by using sticky notes to group the written codes and merge overlapping ones. Secondly, we made a digital thematic map of grouped codes to construct themes and link interpretations. The authors ensured to possess flexibility throughout this process and discussion resolved any disagreements. |
| Reviewing themes | Individually the authors analysed the themes against the raw data to examine if they accurately reflect the data. This process ensured there was no missing themes, and that the themes exist in the original data. Collectively the authors recorded how they understood the themes and how the data supports them with the collating of transcript excerpts that accompany the associated theme. The authors expanded the thematic map with how the identified themes are directly relevant in answering the research question to ensure they are not too broad. |
| Defining and naming themes | The authors collectively described the themes in detail with acknowledgement to the properties and specificity of the theme. All theme names are raw data excerpts (participant quotes) to ensure the definitions are wholly reflective of the original data. The authors defined the themes in alignment with the research aims and objectives and noted how the themes will reflect on the research outcomes. |
| Writing for chosen journal | The authors individually produced a research report which fully encapsulates all of the data and ‘tells the story’ of the qualitative findings consistently. During the writing process the authors reflected on the steps used to ensure a logical format. |
